# Pediatric Systemic Lupus Erythematous in COVID-19 Era

**DOI:** 10.3390/v15020272

**Published:** 2023-01-18

**Authors:** Ancuta Lupu, Ingrith Crenguta Miron, Cristina Gavrilovici, Anca Adam Raileanu, Iuliana Magdalena Starcea, Ileana Ioniuc, Alice Azoicai, Adriana Mocanu, Lacramioara Ionela Butnariu, Felicia Dragan, Vasile Valeriu Lupu

**Affiliations:** 1 Pediatrics, “Grigore T. Popa” University of Medicine and Pharmacy, 700115 Iasi, Romania; 2 Mother and Child Medicine Department, “Grigore T. Popa” University of Medicine and Pharmacy, 700115 Iasi, Romania; 3 Faculty of Medicine and Pharmacy, University of Oradea, 410087 Oradea, Romania

**Keywords:** systemic lupus erythematosus, COVID-19, vaccine, child

## Abstract

Pediatric systemic lupus erythematosus is a chronic autoimmune disorder with a highly variable course and prognosis. It results in functional abnormalities in the immune system due to intrinsic factors and the use of immunosuppressive therapies associated with underlying comorbidities seem to increase the risk of severe COVID-19 and poor outcomes of the disease in pediatric systemic lupus erythematosus (SLE) patients. The aim of this review is to obtain a better understanding of the existing link between this new viral infection and pediatric lupus. We have analyzed the characteristics of newly diagnosed cases of pediatric SLE following COVID-19 which have been reported in the literature and which describe the impact that COVID-19 has on patients already suffering with pediatric SLE.

## 1. Introduction

Systemic lupus erythematosus (SLE) is a chronic autoimmune condition distinguished through its multiorgan involvement and variable severity. Childhood-onset SLE denotes a variant of the disease with an onset within 18 years of age, with a mean age of 11–12 years [1,2]. Pediatric SLE is a rare disorder illustrating 10–20% of all SLE cases. Its incidence stands for 0.3–0.9 per 100,000 patient years and a prevalence of 1.89–25.7 per 100,000 children [3]. Acknowledged since the 13th century, as has science evolved, the characterization of the disease has undergone many updates [4]. 

Even though SLE has been a subject of research for many years, its etiology remains unknown. Its emphasis on several factors, such as genetic, immunological, hormonal and environmental factors played an important role in the underlying mechanisms of the disease. Viral infection seems to generate a substantial immune response concerning both innate and adaptive immunity, a response that is capable of important damage when dysregulated [5,6,7]. A literature search suggests that a link might exist between COVID-19 and the onset of certain autoimmune diseases, but evidence regarding pediatric population is scarce. The aim of this review is to characterize the relationship between pediatric SLE and the new coronavirus disease (COVID-19). We have analyzed the features of newly diagnosed cases of pediatric SLE following COVID-19 that have been reported in the literature and, here, we have described the impact that COVID-19 has on patients already known to be suffering with pediatric SLE in terms of disease epidemiology, risk factors and COVID-19 vaccine safety and related events. An extended amount of time utilized for follow-ups after COVID-19 infection is required to better understand the effects of COVID-19 and pediatric SLE.

## 2. Epidemiology and Risk Factors for COVID-19

Following the COVID-19 pandemic outbreak, severe acute respiratory syndrome coronavirus 2 (SARS-CoV-2) infection became a subject of interest for many researchers, with a huge number of studies reporting different aspects of the disease. Most available data is related to adults, as children infection rates seem to be disproportionally lower [8,9]. Children tend to be asymptomatic or develop mild forms of the disease, resulting a low rate of testing and thus in the potential underestimation of the impact/incidences [8]. The number of affected children varies as the pandemic evolves, depending on testing recommendation and the dominant strain of the virus [10,11].

Although low in number, severe cases are present in the pediatric population, impacting their state of well-being. The guidelines of the Centers for Disease Control and prevention (CDC) reported that the risk of developing severe COVID-19 for pediatric population was higher in neonates, cases of prematurity in young infants, underlying comorbidities such as obesity, diabetes, heart disease, neurologic disease, chronic lung disease, asthma and immunocompromised status (patients undergoing chemotherapy, used immunosuppressant drugs, had immunodeficiency or a hemato-oncologic disorder) [12].

Patients with systemic autoimmune disorder seem to present more severe COVID-19 forms, although the reason behind the increased severity of COVID-19 in this category of patients is still unclear. Studies suggested that the rise in inflammatory markers seen in COVID-19 could be a potential mechanism explaining the phenomena. Patients with systemic autoimmune diseases already have exaggerated immune responses. When associated with COVID-19, their immune response to pro-inflammatory markers becomes further disproportional, leading to a heightened state of COVID-19 infection [13].

Since the pandemic outbreak, individuals affected by SLE have drawn a certain level of attention from the medical community. They are at the increased risk of developing severe forms of SARS-CoV-2 infection, with frequent complications related to the viral disease and often experiencing flare-ups during and after SARS-CoV-2 infection. The presence of comorbidities, organ related damages, intrinsic immune failure and immunosuppressive treatment amplify the concern of medical practitioners related to these patients [14,15]. The immune dysregulation of the interferon pathway encountered in SLE is another reason of concern, mainly because of its role in the innate immune response during the new coronavirus infection [14,15,16]. 

Data from the COVID-19 Global Rheumatology Alliance (C19-GRA) has suggested that SLE patients may be at higher risk of hospitalization from COVID-19 compared to those with other rheumatic diseases [17,18]. Children with rheumatic disease in clinical remission may not be considered as a risk population for severe COVID-19 [15]. Patients with SARS-CoV-2 infection who are known to have a history of autoimmune rheumatic disease and undergoing immunomodulatory treatment are not at high risk of developing severe forms of COVID-19 or complications [19]. The results of Haslak et al. suggested that patients with childhood onset rheumatic diseases, even if treated with immunosuppressive medication might have an asymptomatic SARS-CoV-2 infection, presenting similarly to healthy individuals [20]. According to current studies, some of disease modifying antirheumatic drugs (DMARDs) used by SLE patients have certain effects on COVID-19, influencing the risk of acquiring the infection, disease progression and the immunogenicity of vaccines. Due to their different mechanism of action, DMARDs should be evaluated separately [3]. 

Several SLE adult studies have linked the use of glucocorticoids (GCs), rituximab, cyclophosphamide and presenting an untreated or active form of the disease with severe forms of COVID-19 and poorer outcomes of the infection. GCs use in SLE patients is correlated with severe results in SARS-Cov-2 infection, despite their dose or disease activity [14,21,22]. However, despite the risks associated with GC, most guidelines recommend appropriate GC use without abrupt discontinuation [23]. Disease control in SLE remains the main goal of medical providers, all the more so as uncontrolled disease is a factor for unfavorable COVID-19 outcomes [16].

In children with immune rheumatic disease (IRD), Udaondo et al. reported that patients with active disease and GCs use had an increased risk of COVID-19, similar to the risk described in adults [15]. Rituximab use in adult patients with IRD is linked to a higher rate of hospital admission and death due to COVID-19 [16,24]. As for pediatric patients treated for IRD with Rituximab, the usage has no impact on COVID-19 hospitalization or mortality [25]. Marques et al. suggested that cyclophosphamide treatment might be associated with poor COVID-19 outcomes, whereas long-term anti-TNF drugs may offer protection against infection [26]. Another adult study came to the conclusion that anti-TNF medication cannot be considered a risk factor for severe COVID-19 [27]. Untreated patients’ risk comes from the associated underlying immune dysregulation, while patients with the active form receiving treatment bring together the immune dysregulation and the immune suppression resulting from immunosuppressive therapies. Untreated SLE patients, those with moderate or high disease, actually seem to be associated with more severe outcomes than patients on remission. Patients using belimumab also demonstrated more favorable outcomes [14,28,29]. Methotrexate use in SLE patients was not associated with unfavorable outcomes of COVID-19 [30]. Chronic kidney disease, a frequent complication in SLE, has a strong connection with a negative prognosis in COVID-19 [14].

At the beginning of the pandemic, hydroxychloroquine (HCQ) was considered to be an effective component of COVID-19 treatment [31]; HCQ was used not only as a treatment but also for disease prevention, and this led to a shortage of drugs among patients who were using HCQ as long-term treatment [31]. Ferreira et al. suggested that long-term treatment with HCQ might protect against COVID-19. Patients under chronic use of HCQ have recorded lower SARS-CoV-2 infection rates compared to the general population [32]. However, recent studies evaluating the impact of chronic HCQ use on SARS-CoV-2 infection rates and hospitalization risk and its mortality-reducing effect found no significant evidence [30,33,34,35]. The excessive prescription of HCQ during the pandemic has ended, for which researchers cite the lack of evidence supporting the positive role of HCQ in COVID-19 as well as the associated risk of arrhythmias when used in combination with azithromycin [16].

## 3. Pediatric SLE Onset following COVID-19

COVID-19 infection can trigger the onset of different autoimmune disease in genetically predisposed individuals [6,36]. Even though the underlying mechanism is still unknown, patients with COVID-19 have been shown to have an increase in the general inflammatory markers (LDH, ferritin, creatinine, C3 and C4) as well as more specific ones such as the number of lymphocytes, CRP and IL-6. A study on 40 adult patients with COVID-19 reported that 57.5% of the patients were positive for antinuclear antibodies (ANA) and 25% for antineutrophil autoantibodies (ANCA). Anti-Saccharomyces cerevisiae antibodies (ASCA) IgA was present in 25% of them, ASCA IgG in 17.5% and anti-Cardiolipin antibodies were positive in 12.5% of patients. These results support the idea that COVID-19 is related to autoimmunity [7].

Recent studies have also shown that patients with a critically severe form of COVID-19 infection have increased B-cell activation with secondary increased antibody secreting cell lines [28,36,37,38]. Interferons are thought to play an important role in the development of autoimmune diseases after COVID-19 infection; they are involved in producing adequate response to pathogens and damaged cells in the body. Interferons can be divided into three separate types (type I, II and III), related to the structure and the receptor associated with them [38]. IFN-α and IFN-β are reunited in type I category. Their production is usually induced by viruses, and they affect the release of pro-inflammatory cytokines. Type I interferon responses seem to be strong in relation to COVID-19 infections [38]. An increased expression of IFN-α has been observed in many patients with autoimmune pathology, especially in SLE patients [20]. Over time, SLE onset has been associated to previous viral exposure such Epstein–Barr virus (EBV), parvovirus, cytomegalovirus (CMV), hepatitis C virus and, more recently, SARS-CoV-2 [39,40,41].

Although rare, we thought that analyzing the existing case reports related to pediatric SLE onset following COVID-19 might add value to current medical knowledge. PubMed, Scopus and Google Scholar were searched for relevant publications from December 2019 (when SARS-CoV-2 was first reported) to 15 November 2022. Publications were searched specifically using the terms: “severe acute respiratory syndrome coronavirus 2”, “SARS-CoV-2”, “Coronavirus”, “COVID-19”, “pediatric Lupus Erythematosus, Systemic”, “childhood Lupus Erythematosus, Systemic”, “juvenile Lupus Erythematosus, Systemic”, “Lupus Erythematosus, Systemic, juvenile-onset”. In addition, the references and related citations for the resulting articles were also reviewed for inclusion. Publications were excluded if they included patients 18 years of age and above. Four articles were identified from the citations of analyzed articles, describing four clinical cases of pediatric SLE onset after SARS-CoV-2 infection. The following data were extracted from the studies: age, sex, COVID-19 diagnosis method, days from SARS-CoV-2 infection to first symptom attributable to SLE, clinical feature, laboratory and imagistic findings and received treatment, all summarized in Table 1.

Patients were 11 to 13 years old. Three patients were females. None of them had significant medical history. The diagnosis of COVID-19 was confirmed by real time polymerase chain reaction (RT-PCR) in three cases. One case relied on clinical features for diagnosis as the serology was uncertain. In one patient, the manifestations of SLE began during the acute phase of COVID-19 with moderate illness and hospital admission. In the other three cases, the SLE specific symptomatology appeared 7, 21 days and 2 months after SARS-CoV-2 infection, respectively. All of them presented severe illness requiring hospital admission. Immunological studies showed positive ANA (4 cases), hypocomplementemia (4 cases), positive anti-dsDNA antibodies (4 cases), positive anti-Ro/SSA (2 cases), positive anti-histone (2 case), positive anti-RNP (1 case), and none of them had positive antiphospholipid antibodies, positive anti-La/SSB or anti-β2-glycoprotein I antibodies. Concerning management for SLE activity, all patients underwent corticosteroids (either methylprednisolone or dexamethasone or prednisone), and hydroxychloroquine was added in three patients and azathioprine in one. Other treatments included cyclophosphamide in a patient with renal involvement and plasmapheresis in one patient associating diffuse alveolar hemorrhage. In relation to the clinical course, all patients achieved disease remission.

Cases of SLE after COVID-19 are uncommon. A few SLE adult cases triggered by SARS-CoV-2 infection are also reported in the literature [44,45,46]. The presence of SARS-CoV-2 antibodies and the absence of antibodies for other viruses in all reported patients indicates a link between COVID-19 and SLE. Nevertheless, further studies are required in order to clearly identify the role of COVID-19 in SLE pathogenesis.

## 4. Pediatric SLE and COVID-19 Vaccines

As for COVID-19 vaccination guidelines as they pertain to children, recommendations vary between sources. The CDC recommends COVID-19 vaccination for all children aged 6 months and older. Children aged 6 months to 4 years can receive up to three doses, while those aged 5 years or older can receive the three-dose vaccine scheme and an additional booster dose [47]. The CDC suggests that people moderately or severely immunocompromised should receive an extra primary series dose if receiving the Moderna or Pfizer-BioNTech series. [48]. The WHO is more restrictive and recommends children aged 5 to 15 to receive two vaccine doses. Children with comorbidities and high risk of severe COVID-19 can benefit from the vaccination scheme alongside other high priority category of individuals [49], whereas the National Institute for Health and Care Excellence recommends vaccination for children over 5 years old who meet certain conditions, such as immunosuppression; depending on their clinical status, they can receive up to two doses [50].

Pediatric patients with SLE display a high variability regarding their underlying disease in terms of clinical symptoms, severity, treatments and associated comorbidities. However, evidence concerning the mRNA COVID-19 vaccine suggest that, in SLE pediatric patients, the benefits seem to outweigh the associated risk [51,52].

Despite various numbers of studies regarding the efficacy of SARS-CoV-2 vaccines, there is still a lack of data regarding their use in patients with rheumatic disease, especially pediatric SLE. Since there are many categories of patients for whom general vaccination indication and guidelines do not apply, different scientific societies have developed recommendations for SARS-CoV-2 vaccination of certain groups of patients [23]. 

The American College of Rheumatology (ACR) has elaborated some guideline statements referring to the SARS-CoV-2 vaccination of patients with autoimmune and inflammatory rheumatic diseases (AIIRD). AIIRD patients are considered at high risk for COVID-19 and should be prioritized for vaccination. From the perspective of the general population, the only contraindication to the COVID-19 vaccine is a known allergy to a vaccine component. It is recommended that patients with AIIRD should undergo vaccination during a disease remission period. In patients with active disease forms, the severity of the disorder should be taken into consideration when evaluating the vaccination possibility; patients with active but non-life-threatening pathology should undergo vaccination, whereas patients with life-threatening disease should postpone vaccination until disease control is achieved. Stable and low disease activity patients are recommended to receive vaccination. When evaluating the response to COVID-19 vaccination, clinicians should consider the fact that many patients under systemic immunomodulatory treatment would have a lower level of antibodies, only offering protection for a shorter period of time. AIIRD patients receiving any immunosuppressive or immunomodulatory therapy other than hydroxychloroquine monotherapy, with a completed vaccination scheme of two-dose mRNA series, should receive an additional vaccine dose ≥28 days after the administration of the second dose. As for AIIRD patients not taking any immunosuppressive drugs, ACR recommends starting the vaccination scheme and receiving the first dose before initiating the immunosuppressive therapy. Unvaccinated patients should receive either of the mRNA vaccines, rather than the Johnson & Johnson vaccine. However, the ones who received a one-dose J&J COVID-19 vaccine or who previously completed the mRNA COVID-19 vaccine scheme and require a booster dose should be given an mRNA vaccine supplemental dose. Monoclonal antibody therapy should be given to patients with an increased risk for poor outcomes of SARS-CoV-2 infection, both as prevention and treatment. Following COVID-19 vaccination, there has been documented a risk for flares, disease worsening or new-onset autoimmunity, but for most AIIRD patients, the benefit of vaccination outweighs this risk [23]. The ACR Vaccination Taskforce and the Paediatric Rheumatology European Society recommended prioritizing COVID-19 vaccination for patients with AIIRDs [23,51].

Immune responses to SARS-CoV-2 vaccines involve both humoral and cellular components of adaptive immunity. Viral infections trigger type I IFN production as well as B cell activation, two mechanisms frequently altered in SLE patients and, hence, likely to interfere in their immunological response to vaccines [53]. 

Several studies have been conducted in order to assess the immune response in patients with SLE after COVID-19 vaccination. Immunocompromised adults have demonstrated a suboptimal response to the primary two-dose COVID-19 vaccine series with higher hospitalization rates and greater severity of disease [54,55,56,57]. The analysis of Marra et al. reported that the two-dose COVID-19 vaccine efficacy was 70.4% in immunocompromised adults, with only 63% developing anti-SARS-CoV2 spike protein IgG antibodies compared to 99.1% in the healthy population [58]. A pediatric study reported that most immunocompromised children mount a humoral and cellular immune response to the two-dose COVID-19 vaccine series, a response that is significantly augmented after receiving the third vaccine dose [59].

Izmirly et al. reported a low humoral response in SLE patients after BNT162b2 vaccine administration, possibly as a result of immunosuppression. When compared to healthy subjects, 1/3 of patients displayed lower levels of immunoglobulin G (IgG) antibody against SARS-CoV-2 spike protein receptor binding domain (RBD) [60].

A study conducted in adults with SLE evaluated the neutralizing antibodies (nAB) levels induced by SARS-CoV-2 vaccine after 3 to 6 months from the moment of administration. SLE patients with no associated treatment had nAB similar to healthy control individuals [52]. In contrast, Mehta et al. reported that in SLE patients, the efficacy of COVID-19 vaccination is lower than in the general population, especially for those on MMF, RTX, GC and inactivated vaccines [16]. Similarly, Garcia-Cireira et al. reported that GCs and Rituximab use had a negative influence on medium-term response of SLE patients to SARS-CoV-2 mRNA vaccines, while HCQ use had no impact on nAB levels, compared to control [53]. In contrast, Zheng et al. reported that GCs in low doses had negligible effect on vaccine immunogenicity, while the results in HCQ and Rituximab use were close to ones reported by Garcia-Cereira et al. [21,53].

Many studies have reported on the safety of vaccines in adolescent and adult patients known with SLE [20,61]. Several plausible risk factors for systemic rheumatic disease (SRD) flares should be considered in relation to flares after vaccination. First, comorbidities and demographic factors, which are also risk factors for developing RA and other SRDs, or are associated with disease activity, may be related to flares after vaccination. Beyond baseline characteristics, cessation of SRD therapies could contribute to disease flares [62]. In their study on adult SLE, Mok et al. reported that patients with active serology and a history of arthritis and discoid skin lesions are more likely to develop disease flares after receiving the COVID-19 vaccine [63].

An international study evaluated the appearance of flares requiring a change in treatment following COVID-19 vaccination in 4.9% of adults with systemic rheumatic disease. Compared to others rheumatic disorders, SLE was associated with higher odds of flare [62]. 

Zavala-Flores et al. found a 27% rate of SLE reactivation episodes in adults immunized with BNT162b2 vaccine against SARS-CoV-2 via a two-dose vaccination scheme. Articular manifestations were predominating symptoms (85.1%), followed by dermal flares (18.5%) [64]. Similarly, Sun et al. reported a 22.2% rate of SLE flare after vaccination [65].

Izmirly et al. described up to 11.5% of disease reactivation episodes in adult patients with SLE after SARS-Cov-2 immunization [60]. Another study reported 3% episodes of SLE flare in adults following SARS-CoV-2 vaccination [61]. Barbhaiya et al. data suggested > 91% of SLE patients did not flare post-SARS-CoV-2 vaccination, and most flares were mild or moderate. Seventy-four percent reported a vaccine related AE [66].

In a single-center study evaluating the safety of COVID- 19 vaccination in pediatric SLE, adverse events were noted in four of sixteen patients (25%), and none were severe. Two patients reported moderate flares after vaccination [20]. A prospective study evaluating the safety and immunogenicity of mRNA-based anti-SARS-CoV-2 vaccines in a cohort of adolescents with juvenile-onset AIIRDs reported minimal or no side effects in 96.7% of patients. Post-vaccination indices of disease activity remained stable with a mean change of −0.2 (s.d. 2.9) for 10 SLE patients [67]. Similar results of vaccine safety were reported by Akgün et al. in their study [68].

According to recent studies, reported flare rates after vaccination in adult SLE population vary from 3% to 25% [62,63,64,65,66]. Numbers are higher in studies based on patients’ self-assessment reported flares via telephonic survey or questionnaire, while medical confirmed flares remained low. However, adult reported flares remain higher than those corresponding to pediatric population, since the number of pediatric patients included in studies is very low and there is a lack of data referring to pediatric SLE patients and their experience with COVID-19.

Beside adverse reaction and disease flare, new-onset SLE among previously healthy patients vaccinated against COVID-19 has recently emerged sporadically. All reported cases were adults; none related to children [69]. Two mechanisms have been suggested to explain the association between the foreign antigen (of the vaccine) and autoimmunity: molecular mimicry and activation of antigen-presenting cells toll-like receptors (TLRs) [6,70]. COVID-19 vaccination is not the first vaccine associated to SLE onset, as previous studies report similar cases after hepatitis B and human papillomavirus vaccine administration [71,72].

There is little evidence available in the medical literature regarding the course of COVID-19 in pediatric patients and even less studies about COVID-19 in SLE pediatric patients. In this particular issues, clinical investigators face many challenges when conducting research involving children. The high number of asymptomatic COVID-19 pediatric cases and the parental reluctance towards having their children enrolled in a medical research study in the current pandemic situation are important facts explaining the scarce published literature. Although it is well known that children are not miniature adults, extrapolating data from adult studies is a current practice used in the absence of high-quality evidence in pediatric population. In the present research paper, we considered it appropriate to fill the current scientific gaps related to pediatric SLE and COVID-19 with data from adult studies or data of patients with IRD until new evidence emerges. Most studies on IRD include SLE patients in their population. Results can be used to generate future research specific to pediatric lupus.

## 5. Conclusions

Pediatric SLE is a long-standing disease that acquires new features in the COVID-19 pandemic context. Patients with pediatric systemic lupus erythematosus are associated with an increased risk of viral infection and poor outcomes with SARS-CoV-2 infection. COVID-19 vaccination is considered safe, and it is recommended for pediatric lupus patients, although vaccine efficacy might be influenced by immunosuppressant therapy. Patients with SLE can experience disease flares post vaccination. Most pediatric recommendations rely on adult guidelines, as the pediatric lupus literature related to COVID-19 is scarce. New-onset pediatric SLE cases have been reported following COVID-19. The possible role of SARS-COV-2 in triggering pediatric SLE in genetically susceptible individuals needs further study as there might be a link between autoimmune diseases and COVID-19.

## Figures and Tables

**Table 1:** SLE patients clinical, biological, imagistic and therapeutic characteristics.

References	Age/ Sex	COVID-19 Confirmation Test	Clinical Features	Laboratory Findings	Additional Testing	Treatment
Bettiol et al., 2020 [41]	11/F	Negative RT-PCR, Ig M Ig G- 14 U/ml	Left palpebral oedema facial butterfly cutaneous eruption atypical papules on arms and forearms cervical adenopathy stiffness myalgia dysphagia ageusia anosmia abdominal pain pyrexia	Neutropenia Mild normocytic anemia Elevated ESR Hypergammaglobulinemia Positive ANA 1/320, anti-ds DNA ab, anti-nucleoprotein ab (nRNP/Sm, Sm, SS-A60, Ro-52, AMA M2, SRP, Rib P protein, histone, nucleosome), ANCA (1/160 dilution) Negative anti MPO, PR3 Low C3, C4 Negative hepatitis B, C, HIV, EBV, CMV, parvovirus B19, Herpes Zoster, measles, toxoplasmosis	Thoraco-abdominal CT scan: axillar and cervical adenopathy ECG: first-degree atrioventricular block Normal cardiac and abdominal ultrasound Normal cerebral CT scan Skin biopsy: connective tissue disease	IV MP one bolus-changed to Oral MP/daily HDQ daily
Maram et al., 2021 [42]	12/M	Positive RT-PCR 7 days before presentation	Facial puffiness pedal oedema Itchy, vesiculo-bullous skin eruption over the trunk	Gross urinary proteinuria elevated urine protein creatinine ratio Low serum albumin High cholesterol levels Positive ANA 1/80, anti-ds DNA 1/10 Low C3, C4 levels Normal ASO titer	Skin biopsy: sub-epidermal bullae with eosinophilic infiltrate	OS diuretics-poor response
					7 days later Renal biopsy: class V nephritis without tubular atrophy	7 days later IV cyclophosphamide 750 mg/m^2^ every 2 weeks
Rauf et al., 2022 [43]	12/F	Negative RT-PCR Positive IgG 21 days before presentation	Progressive fatigability Severe palor 12 h after admission Acute-onset headache, delirium, signs of meningeal irritation Swelling in the left knee joint with tenderness	Autoimmune haemolytic anaemia Peripheral smear: polychromasia with nucleated RBCs High levels of CRP, ESR, D-dimer Positive direct and indirect Coombs test with monospecific anti-human globulin card test showing anti-IgG and anti-C3d-positive	Cerebral fluid analysis: cells: 5 (all lymphocytes), glucose—100 mg/dL protein—20 mg/dL-bacterial meningitis Normal echocardiography	Neuroprotective measures antibiotics IV steroids (DXM 0.15 mg/kg/ dose 6 hourly) IV immunoglobulin (2 g/kg over 24 h) Aspirin (treatment protocol for MIS-C) One aliquot/unit of packed red blood cell transfusion
				2 days later Positive ANA, anti-ds DNA ab, anti-histone ab Low C3, C4 Negative anti-phospholipid ab, anticardiolipin and anti-beta2-glycoprotein ab	2 days later Bone marrow smear: erythroid hyperplasia with lupus erythematosus (LE)	2 days later IV steroids were changed to OS (prednisolone) HCQ
Asseri et al., 2022 [2]	13/F	Positive RT-PRC 2 months before presentation	Acute respiratory failure	Anemia Negative direct Coombs test Positive indirect Coombs test Negative EBV, parvovirus B19, CMV, retroviruses, and HIV Positive ANA, anti-ds DNA ab Low C3, C4 Negative antiglomerular basement membrane ab, rheumatoid factor, antineutrophil cytoplasmic ab, proteinase-3 ab, anti-cardiolipin IgG ab, beta-2 glycoprotein ab, and anti-Smith ab	Chest X-ray: bilateral consolidation silhouetting bilateral hemidiaphragm and cardiac borders Chest CT scan: bilateral consolidation and ground-glass appearance Normal echocardiography Flexible bronchoscopy- BAL: blood-stained fluid and yielded abundant hemosiderin-laden macrophages	IV bolus MP broad-spectrum antibiotics supportive measures- poor outcome 6 sessions of plasma exchange with maintenance MP (2 mg/kg/d) *Discharge:* OS (prednisolone) HCQ AZA

## Data Availability

Data sharing not applicable.
